# Quantification of Celiac Disease Severity Using Video Capsule Endoscopy: A Comparison of Human Experts and Machine Learning Algorithms

**DOI:** 10.2174/1573405619666230123110957

**Published:** 2023-06-02

**Authors:** Stefania Chetcuti Zammit, Mark E. McAlindon, Elliot Greenblatt, Michael Maker, Jenifer Siegelman, Daniel A. Leffler, Ozlem Yardibi, David Raunig, Terry Brown, Reena Sidhu

**Affiliations:** 1 Academic Unit of Gastroenterology and Hepatology, Sheffield Teaching Hospitals NHS Hospital Trust, Sheffield, S10 2JF, UK;; 2 Invicro, a Konica Minolta Company, Boston, MA, USA;; 3 Takeda Pharmaceuticals Inc. Co., Cambridge, MA, USA;; 4 Department of Infection, Immunity and Cardiovascular Disease, University of Sheffield, Sheffield, S10 2RX, UK

**Keywords:** Celiac disease, machine learning algorithm, quantitative analysis, small bowel imaging, video capsule endoscopy, MPEG

## Abstract

**Background:**

Video capsule endoscopy (VCE) is an attractive method for diagnosing and objectively monitoring disease activity in celiac disease (CeD). Its use, facilitated by artificial intelligence-based tools, may allow computer-assisted interpretation of VCE studies, transforming a subjective test into a quantitative and reproducible measurement tool.

**Objective:**

To evaluate and compare objective CeD severity assessment as determined with VCE by expert human readers and a machine learning algorithm (MLA).

**Methods:**

Patients ≥ 18 years with histologically proven CeD underwent VCE. Examination frames were scored by three readers from one center and the MLA, using a 4-point ordinal scale for assessing the severity of CeD enteropathy. After scoring, curves representing CeD severity across the entire small intestine (SI) and individual tertiles (proximal, mid, and distal) were fitted for each reader and the MLA. All comparisons used Krippendorff’s alpha; values > 0.8 represent excellent to ‘almost perfect’ inter-reader agreement.

**Results:**

VCEs from 63 patients were scored. Readers demonstrated strong inter-reader agreement on celiac villous damage (alpha=0.924), and mean value reader curves showed similarly excellent agreement with MLA curves (alpha=0.935). Average reader and MLA curves were comparable for mean and maximum values for the first SI tertile (alphas=0.932 and 0.867, respectively) and the mean value over the entire SI (alpha=0.945).

**Conclusion:**

A novel MLA demonstrated excellent agreement on whole SI imaging with three expert gastroenterologists. An ordinal scale permitted high inter-reader agreement, accurately and reliably replicated by the MLA. Interpreting VCEs using MLAs may allow automated diagnosis and disease burden assessment in CeD.

## 1 INTRODUCTION

Celiac disease (CeD) is a chronic, immune-mediated disorder triggered by dietary gluten in genetically predisposed individuals, with a prevalence of approximately 1% in many populations worldwide [1, 2]. Small intestinal enteropathy after gluten ingestion is considered a hallmark of CeD [1]. Disease pathology is often non-continuous and patchy, with villous damage throughout the small bowel, although this is generally more pronounced in the proximal region, with healing known to occur in the more distal segments [3-5]. Effective mechanisms are required to monitor treatment response and aid disease management in order to reduce the impact of CeD and long-term disease complications [1, 6].

Duodenal histology, together with positive celiac serology, represents the standard for diagnosis of CeD [7]. Serologic testing, such as for anti-tissue transglutaminase, is insufficient for monitoring and has been demonstrated to be insensitive in detecting persistent small intestinal mucosal injury [8]. Other established methods for CeD monitoring, such as duodenal biopsy and endoscopy, do not allow a review of the entire small bowel, are invasive, and may require sedation, which carries additional risks [9-12]. Furthermore, upper gastrointestinal endoscopy and duodenal biopsy are costly and require careful review by expert pathologists, with specimen preparation and interpretation processes poorly standardized in clinical practice [10, 12]. Therefore, improved techniques would be helpful in combatting inaccurate diagnosis of CeD and the challenges in monitoring the effectiveness of therapy, and would provide a more comprehensive picture of disease that can help to guide patient management.

The use of video capsule endoscopy (VCE) is well established for the detection of CeD, in addition to other gastrointestinal diseases [13-15]. In contrast to the sparse sampling of a few microns of tissue provided by endoscopic biopsy, VCE offers an attractive, minimally invasive method of evaluating the entire bowel, albeit at a macroscopic level, and could potentially play an important role as an adjunctive method in the monitoring and management of CeD and, in the future, diagnosis. On a broader scale, VCE has the potential to be usefully employed in a range of circumstances, including outpatient or ambulatory settings.

Despite the primary benefit of assessing the entire small intestine (SI), several limitations have been historically associated with VCE, including inter-reader reproducibility, required expertise, and the associated workload burden. Concerns exist regarding the reliability of VCE for CeD evaluation, most notably about the varying degree of reader experience/ability to detect and classify the subtle features apparent during VCE [16-18]. Reader disagreement is particularly problematic in studies assessing specific qualitative findings, such as scalloping, fissuring, atrophy, and/or mosaicism. In addition, review of VCE images is time-consuming for gastroenterologists, and reimbursement can be low considering the time taken for image analysis [19, 20]. The subject of this analysis is an automated method of evaluating villous atrophy in CeD developed to improve the reproducibility of VCE, which is hypothesized to be important when assessing the benefit or harm of an intervention or therapy, and to reduce the variability and time burden associated with the interpretation of an entire VCE video [21, 22]. With this work, we offer a new method for assessing the whole small bowel burden of CeD.

The use of artificial intelligence and machine learning methods to interpret endoscopic images has been explored in small bowel disease, in which data have demonstrated that deep learning-based algorithms can identify clinically significant and normal variant gastrointestinal abnormalities with higher levels of sensitivity and specificity, and significantly shorter reading times, than conventional analysis by gastroenterologists [23]. Accordingly, artificial intelligence models are rapidly emerging as breakthrough technologies in endoscopy and offer the opportunity to revolutionize the use of VCE in clinical practice [12, 24-27]. Currently, there are no widely accepted standardization or quantification methods for measurement of small bowel villous damage in CeD.

In this study, we evaluated the objective assessment of villous damage in patients with CeD by expert human readers using an unbiased VCE scoring tool and disease assessment performed *via* a machine learning algorithm (MLA). As an exploratory objective, we also sought to assess the association between VCE assessment results and clinical parameters, including physician global assessment, serology, and biopsy results.

## 2 MATERIALS AND METHODS

### 2.1 Participants

Patients aged at least 18 years with histologically proven CeD underwent clinically indicated VCE, using PillCam™ SB 3 (Medtronic, MN, USA). VCE videos were retrospectively identified and randomly selected from the Sheffield Teaching Hospitals NHS Foundation Trust archive. Patients were required to have either a new diagnosis of biopsy-confirmed CeD with positive serology results at the time of diagnosis or established biopsy-confirmed CeD. Patient biopsies were examined by two histopathologists who confirmed the presence of CeD. Patients were excluded if the VCE contained excessive artifacts consistent with poor gut preparation, or an incomplete VCE video. An incomplete VCE video was determined as a capsule endoscopy that did not reach the cecum and therefore did not provide a complete visualization of the small bowel. Further details on patient consent can be found under the ‘Ethics approval’ heading later in this section. This study was sponsored by Takeda Pharmaceutical Company Ltd.

### 2.2 VCE Image Acquisition Procedures

Patients were asked to stay on clear fluids for 24 hours before VCE, and to drink 2 L of Klean-Prep^®^ solution (Norgine Ltd., Uxbridge, UK) the day before the procedure as part of routine clinical practice for all VCE indications.

### 2.3 Data Analysis

#### 2.3.1 VCE Scoring

VCE videos were scored by three expert physicians from the same institution who have each read more than 1000 VCEs, and who assess more than 300 VCEs annually. The same 63 videos were also scored by the MLA. Experts’ scores were collected in an unbiased fashion, by providing randomly ordered video images using a custom VCE sampling tool developed in MATLAB^®^ (MathWorks, Natick, MA, USA). The experts utilized a previously described novel, 4-point ordinal visual assessment scale for evaluating the severity of enteropathy in CeD [28, 29]. Individual frames were scored as 0 (no disease), 1 (mild disease), 2 (moderate disease), or 3 (severe disease).

Expert readers were presented with shuffled frames derived from videos of 5-10 different patients in a single reading session, with frames from all videos in a session interspersed. Experts evaluated dispersed frames until 60 frames had been scored for each video. Expert readers were tasked to assign a score to each frame when they felt confident to do so. However, a short video segment of sequential images preceding and following the single frame was also provided to assist the scoring and decision process.

#### 2.3.2 Machine Learning

##### 2.3.2.1 Training Data

The MLA was trained to score individual frames using an independent training data set of 334 080 frames from 35 patients with biopsy-proven CeD, who underwent VCE as part of their clinical care. In addition, 110 579 frames from 13 patients without CeD who underwent VCE for unrelated symptoms, such as gastrointestinal bleeding or assessment for cancer, were also added to the training data. All training frames were distinct from the VCE frames used in this study, and 5505 frames overall were scored by a technical image analyst based on the celiac enteropathy-villous atrophy scale (CeVASt) [28, 29]. None of the experts were involved in training the technical analyst. Primary validation was performed on an additional 36 VCE test set from 12 patients at three time points.

##### 2.3.2.2 Pre-processing

The last pylorus frame and the first cecal frame were marked and exported as a Moving Picture Experts Group (MPEG) video using PillWeb (Medtronic, Minneapolis, MN, USA). The videos were broken into frames using Fast Forward MPEG [30]. These frames were initially pre-processed by cropping to the 512 × 512 portion of the image frame and converted to grayscale using the geometric mean of the RGB (red, green, blue) color channels. The position along the SI was encoded in 8-bit (0-255) in the corner of the frame.

##### 2.3.2.3 Base Network

A residual network (ResNet), a form of convolutional neural network (CNN) [31], was trained to regress the curve value using a training set with a mean squared error training loss. A curve value was available for all frames, with an average of 9200 frames per video, even if only 60-120 frames were annotated. We used a ResNet50 [31] architecture with 64 3 × 3 filters at the lowest level, and four layers, each with a 3,4,6,3 residual block schedule. The architecture was implemented in Python v3.6 (Python Software Foundation, Wilmington, DE, USA) using ANTsPyNet and TensorFlow as the backend [32].

##### 2.3.2.4 Error Prediction Modification

Once the base layer was trained, a second output was added to the network to estimate the error in the frame prediction. The loss was updated to reflect the sum of the absolute error in prediction (the curve value minus the predicted value) and the absolute error of the predicted error (the error of the prediction minus the predicted error), with the latter term weighted by a factor that increased from 0 to 100 over the training period [33]. The weighting factor amplified the importance of the error prediction in the loss function, and with a factor value of 100, error prediction was the primary driver of loss. Each video was machine analyzed in fewer than 15 minutes using a single NVIDIA^®^ V100 graphics processing unit (NVIDIA^®^ Corporation, Santa Clara, CA, USA), with 32 gigabytes of dedicated random-access memory on an NVIDIA^®^ DGX workstation.

After several days of training on the workstation, the coefficient of determination (R^2^) for the predicted frame severity with the actual severity on eight reserved validation videos was 0.89. The R^2^ for the error prediction was 0.5 for these data (*P* < 0.0001).

##### 2.3.2.5 Ensembled Prediction

Each frame was read by the MLA in the eight different orientations that are possible by vertical and horizontal flipping and rotation of the frame. A (1/error)^2^ weighted average of both the mean celiac severity prediction and the error was calculated, along with the standard deviation of the prediction with orientation.

##### 2.3.2.6 Curve Fit and Post-processing

The individual curve and error estimates were aggregated to generate a curve, along with plots and derived metrics, using a compiled MATLAB^®^ script. The curve fit used a weighted average of frames within 1/9th of the SI, with the weighting of each frame proportional to (1/net error)^2^. The net error for each frame was derived from several sources. The first source of error was estimated from a linear combination of two estimates of frame prediction error: the standard deviation of the changes in prediction with orientation and the weighted average error prediction from the CNN. From the training data, it was found that 0.96 frames that were further from the point of estimation on the curve had decreased predictive power. This value was empirically measured by fitting a difference between a scored frame *versus* their difference along the SI and calculating the slope through zero. This value was measured at 0.84/SI, or a delta of 0.84 in severity for a difference of one SI length between points. The original frames that were scored in the training set were discretized and this source of error had a residual influence on predictions by the MLA. Bootstrapping a uniform distribution between 0-3 showed that the root mean square error due to discretization was approximately 0.289.

Errors were treated as unsystematic, and owing to the high number of frames available for scoring, these sources of error were greatly abated for reads by the MLA. By contrast, a systematic source of error arose from the difference between individual expert reader scores and the true, unseen, severity of CeD. The average among experts was taken as a proxy for this truth, and the error value was estimated from the mean absolute error of the CeVASt score of an individual expert *versus* the average score among 350 frames scored by three experts (0.1875). Finally, the MLA curves were corrected for under-dispersion. No correction for over-dispersion was performed.

Additional figures illustrating the technical aspects of the MLA are provided in Figs. **S1** and **S2**.

#### 2.3.3 Data Handling

After scoring, curves representing CeD severity and position (proximal, mid, and distal) in the SI were fitted to data from each video and for each expert human reader. Individual frame severity and error estimates were aggregated to generate each curve using a compiled MATLAB script. Curve fit used a weighted average of frames within 1/9th of the SI, in which the weighting of each frame is proportional to (1/net error)^2^. The measurement of 1/9th was chosen because it is small enough to show consistent changes within an SI tertile while still providing smooth curves.

Curves were compared at 10 evenly distributed points along the patient’s small bowel. These 10 points were used because they could be identified with no influence on each other with 1/9th of the SI weighting. The mean and maximum values of the curve in the first tertile (first tertile mean and maximum) and the mean curve value for the entire SI were also compared between human readers. Additionally, the mean scores of the frames in the first 5% of the video were also assessed. Frames from the first 5% were sequestered into a separate metric after noticing that some villous atrophy was observed in this region in several non-CeD videos in the training data, potentially due to gastric injury. After the assessment of the expert human readers, average reader curves and derived metrics were compared with the scores generated by the MLA.

Summary plots were also generated to compare average expert reader and MLA celiac severity scores across all patients, for the first tertile mean, first tertile maximum, and the mean value across the entire SI.

#### 2.3.4 Clinical Data

The associated methodology for the assessment of the association between VCE findings and clinical parameters is provided in Supplementary Text **S1**. Further details of the physician global assessment parameters evaluated are provided in Table **S1**.

### 2.4 Statistics

All inter-reader comparisons, and comparisons between human readers and the MLA, were performed using Krippendorff’s alpha for interval data. Krippendorff’s alpha is an index of reliability measuring agreement between any number of raters or observers, that may be used with binary dichotomous or nominal data and continuous data with or without missing values [34]. This permitted the comparison of metrics for more than two readers, and allowed all available pairs of data to be used even if missing for one reader. Krippendorff’s alpha (alpha) values may be interpreted similarly to the kappa statistic, as: 0-0.1, no agreement; 0.2-0.4, fair agreement; 0.4-0.6, moderate agreement; 0.6-0.8, substantial agreement; and > 0.8, almost perfect agreement [35]. For this study, alpha values above 0.8 were considered to indicate agreement among readers [36]. For summary plots, R^2^ was used to compare expert reader and MLA celiac severity score linearity across all patients assessed, with 0 indicating no correlation and 1 representing perfect correlation. Statistical significance was determined at *P* < 0.05.

### 2.5 Ethics Approval

The study protocol was approved by the Yorkshire and Humber Research Ethics Committee (IRAS 232382) and registered with the local research and development department of Sheffield Teaching Hospitals NHS Foundation Trust (registration number STH 19998). All patients provided consent prior to the VCE procedure as part of their usual clinical care. All images used in this study were de-identified. No additional consent was required for the study with the use of de-identified videos, as assessed and approved formally by the Research Ethics Committee.

## 3 RESULTS

### 3.1 Patient Demographics and Disease Characteristics

In total, 63 VCE videos from 63 patients with biopsy-confirmed CeD were included for analysis. Patients had a mean (standard deviation) age of 45.0 (18.3) years at the time of VCE and were mostly female (65.1%; 41 patients). At the time of VCE, 44 patients had a new CeD diagnosis based on duodenal histology, and 19 had established disease. Patients receiving a new diagnosis had not yet begun dietary measures, whereas patients with established CeD had spent an average of 167 months on a gluten-free diet. In all cases, indications for VCE were consistent with local European guidelines. They included the assessment of potential complications (17 patients), the extent of CeD (44 patients), and improvement in response to treatment (2 patients). Additional laboratory data for all patients at the time of VCE are shown in Table **S2**.

### 3.2 Curve and VCE Metrics

**Fig. (1):**
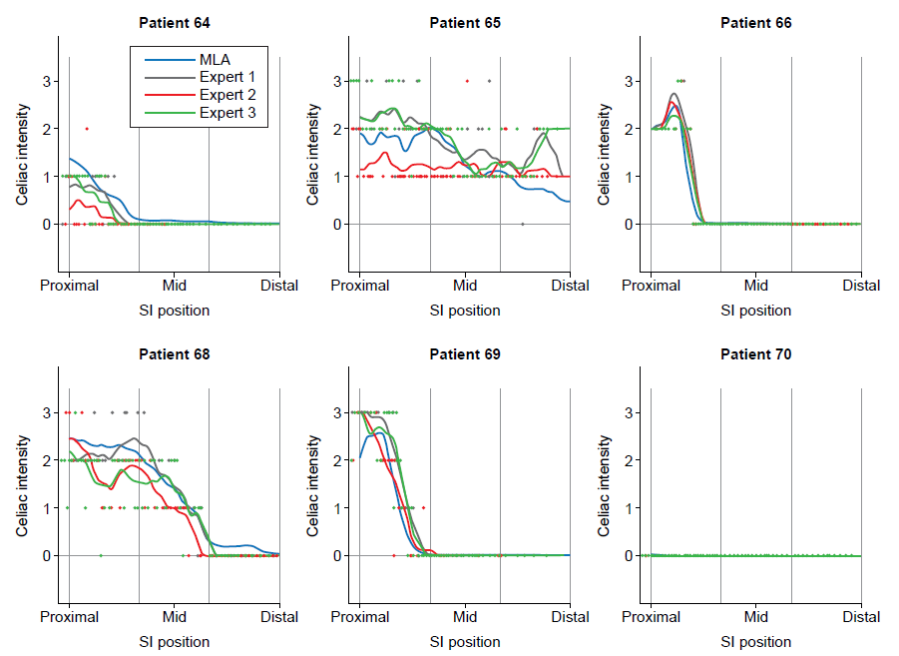
Sample patient celiac severity curves. Individual VCE frames were scored for celiac severity as 0 (no disease), 1 (mild disease), 2 (moderate disease), or 3 (severe disease). The curves generated provide a visual representation of the severity of the disease at a certain location on the y-axis and the disease severity on the x-axis. MLA, machine learning algorithm; SI, small intestine; VCE, video capsule endoscopy.

Celiac severity curves were similar among expert readers (alpha = 0.924), with over 99% within one increment of the recorded celiac severity score selected by other readers (representative celiac severity curves for individual patients provided in Fig. **1**). First tertile mean, first tertile maximum, and mean over the entire SI were also comparable among expert readers, with alpha values of 0.94, 0.87, and 0.95, respectively. The average celiac severity curves generated by expert human readers were similar to those for the MLA, with an overall alpha value of 0.935. In addition, average expert reader and machine-read curves were comparable for first tertile mean (alpha = 0.932 [individual expert reader alpha values ranged from 0.875-0.9^37^]), first tertile maximum (alpha = 0.867 [0.792-0.861]), and the mean value over the entire SI (alpha = 0.945 [0.914-0.9^40^]). On average, expert readers spent 5.4 seconds scoring each frame and 5.5 minutes per VCE video.

**Fig. (2):**
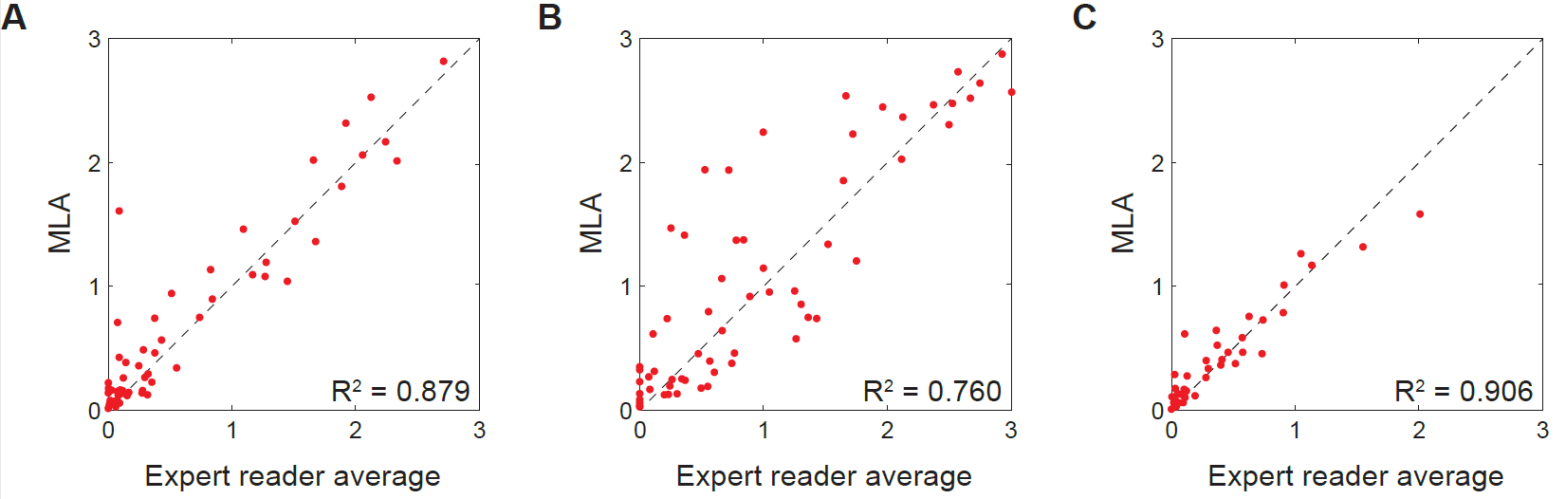
Summary plots comparing average expert reader and machine learning celiac severity scores across all patients for (**A**) the first tertile mean, (**B**) the first tertile maximum, and (**C**) the mean value across the entire small intestine. Video capsule endoscopy frames were scored for celiac severity as 0 (no disease), 1 (mild disease), 2 (moderate disease), or 3 (severe disease). MLA, machine learning algorithm; R^2^, coefficient of determination.

Across all patients, a comparison of the average celiac severity scores provided by the three expert readers showed a strong correlation with scores provided by the MLA. This finding was observed over the first tertile mean (R^2^ = 0.879), first tertile maximum (R^2^ = 0.760) and the mean value across the entire SI (R^2^ = 0.906; Fig. **2**).

### 3.3 Comparison of Expert Reader VCE Findings and Clinical Parameters

Data summarizing results from the comparison of VCE findings with patient serology, physician global assessment, histologic findings, presence of endomysial antibodies, and genetic and refractory status are given in Supplementary Text **2** and Tables **S3-S5**. Overall, little correlation was seen between clinical data and VCE findings.

## 4 DISCUSSION

This study introduces a new method for investigating the whole small bowel burden of CeD. We assessed the ability of machine learning to grade disease severity in CeD quantitatively by evaluating MLA performance in scoring CeD severity against that of three expert gastroenterologists using a commercially available medical device with a favorable safety profile. The MLA performed well in assessing CeD severity and against average data across a number of expert human readers. Across all patients, a strong correlation was observed between celiac severity scores provided by the MLA and the average expert reader scores, with R^2^ values ranging from 0.760 to 0.906 over the different metrics evaluated. Some inter-reader variability was observed even among three clinical experts, and it could be reasonably expected that this variability would be much greater among readers of differing levels of VCE experience in a real-world setting. From the literature, inter-reader agreement is lower among readers with limited experience of VCE than among those highly experienced in VCE interpretation, and the use of more traditional, qualitative scoring techniques with subjective, descriptive terms when interpreting VCE images in CeD can also reduce agreement between readers [16, 17]. In addition to being similarly accurate to expert human readers, the MLA could have even greater value in settings in which readers have less VCE experience and could be akin to introducing an expert reader into every practice.

Studies have previously investigated the use of machine learning methods in the diagnosis and assessment of CeD, including deep learning methods and image processing techniques [12, 27, 37]. Zhou *et al.* took a deep learning approach to distinguish between patients with CeD and healthy individuals, and reported 100% sensitivity and specificity [12, 27]. However, the sample size was small (n = 10), and the patient populations used for machine training (six patients with CeD, five healthy controls) and for evaluation did not all have confirmed CeD [27]. This contrasts with the biopsy-confirmed population in the current study, in which the newly developed 4-point ordinal scale allowed estimation of CeD burden along the entire SI, providing greater insight and specification than global patient classification methods. A lack of strict criteria for patient populations used for training of machine learning methods, as in Zhou *et al.* [27], may limit the ability of this technology to distinguish CeD in populations with concomitant gastrointestinal conditions, and affect the robustness of this study. Additionally, only patients with evidence of CeD in more than 50% of their frames were considered to have CeD, whereas patients may be affected in only one portion of the SI [27]. These factors should be considered in future studies seeking to refine the use of MLAs to interpret VCE images in CeD.

Image processing techniques have also been evaluated in CeD. In a study by Koh *et al.* [37], discrete wavelet transform and nonlinear feature techniques were used to manage VCE images and identify CeD. An accuracy level of 86.47%, and sensitivity and specificity of 88.43% and 84.60%, respectively, were achieved. However, again the sample size evaluated was small (n = 26) and the method required human-engineered features to distinguish between patients with CeD and controls, creating additional complexity and time considerations. Additionally, these algorithms were intended to simply detect CeD, the presence of which is binary, and did not offer a measure of disease severity. This contrasts with the MLA used in our study, which assesses CeD severity using VCE images alone, offering greater ease of use in clinical and research settings.

Although additional studies in larger populations are required to validate the MLA further, the current literature highlights the value of computer-automated diagnosis and evaluation of CeD [12, 27, 37]. The use of machine learning methods to automate VCE image analysis averts the need for lengthy assessments of video examinations by expert human readers, reducing the time and costs associated with investigation and potentially freeing up physicians for other therapeutic procedures [23]. Machine learning methods also increase the throughput and efficiency of image interpretation, as well as quantitatively standardizing the reporting of VCE findings in CeD, potentially facilitating consistency in patient management and care across differing healthcare settings. Machine learning techniques may also aid monitoring of disease pathology in patients with refractory CeD or patients unable or unwilling to undergo more invasive methods of investigation, such as children or the elderly. Because VCE is already widely available, it is well positioned for use as a clinical trial endpoint to monitor treatment effectiveness in many conditions and could potentially provide an opportunity for integration with wearable technology, facilitating remote and more frequent patient monitoring [38]. However, it must be noted that, given the mixed results of current studies, additional analyses of the correlation between histologic and VCE findings are required [17, 18, 39, 40]. Further evaluation of VCE, and the interplay between its findings and aspects of the wider clinical picture in the diagnosis and management of CeD, represent the next steps for this technology and its use in patient care.

Our study has several strengths. The MLA demonstrated a strong performance in assessing CeD severity, comparable with three expert gastroenterologists, despite the complexity of disease features captured in VCE images and their potential variation in severity. Additionally, the analysis was conducted in a large patient cohort with well-characterized disease and varying levels of CeD severity. The quantitative scoring scale allowed for more straightforward and reproducible assessment and improved inter-observer agreement, and readers could view short video segments rather than still images, permitting more detailed and robust analysis. The random shuffling of patient images presented to human readers reduced the possibility of any unknown bias during image evaluation, and readers were blinded to the severity of duodenal histology and CeD serology for each patient, as well as each other’s scores, further reducing bias. However, several study limitations require consideration. Referral bias may be present, because the study was conducted at a single national CeD center in the UK, and may have contributed to the findings of this study. Although MLA training was conducted using limited data and possible referral bias may have contributed to the results, a solid performance was still observed. Additionally, further data are not available regarding reproducibility of MLA performance against a wider range of experts. Not all CeD is evident on VCE, meaning some patients appeared to have no disease despite having a biopsy-confirmed diagnosis. Finally, VCE images, including complications such as enteropathy-associated T-cell lymphomas and adenocarcinoma were excluded from MLA assessment. Further algorithm development and training on wider data sets would be required to allow evaluation of these complications.

## CONCLUSION

Overall, our findings demonstrate that an MLA trained on the videos of 48 patients can accurately and reliably replicate the assessment of CeD severity generated by expert gastroenterologists. Machine learning technologies therefore represent potentially valuable tools that could revolutionize the detection and quantitative assessment of CeD in clinical and research settings. The CeD severity curves provide a visual representation of the overall burden of villous damage, and offer a new, quantitative way to view CeD. This study indicates that this representation has good inter-reader reproducibility, and can potentially also be measured by an MLA. This study demonstrated that VCE can provide a detailed spatial assessment of the severity and extent of small intestinal mucosal damage in CeD. Incorporating this technology in the future may enable automated diagnosis, evaluation, and referral of patients with CeD, particularly in centers where expertise is limited, and therefore ensure appropriate and consistent patient management and care escalation.

## Data Availability

The authors confirm that the data supporting the findings of this study are available within the article and its supplementary materials.

## LIST OF ABBREVIATIONS

CeD: Celiac Disease
CeVASt: Celiac Enteropathy-Villous Atrophy Scale
CNN: Convolutional Neural Network
MLA: Machine Learning Algorithm
MPEG: Moving Picture Experts Group
R^2^: Coefficient of Determination
ResNet: Residual Network
SI: Small Intestine
VCE: Video Capsule Endoscopy

## References

[1] Kelly C.P., Bai J.C., Liu E., Leffler D.A. (2015). Advances in diagnosis and management of celiac disease.. Gastroenterology.

[2] Rostom A., Murray J.A., Kagnoff M.F. (2006). American Gastroenterological Association (AGA) Institute technical review on the diagnosis and management of celiac disease.. Gastroenterology.

[3] Ludvigsson J.F., Leffler D.A., Bai J.C. (2013). The Oslo definitions for coeliac disease and related terms.. Gut.

[4] Lidums I., Teo E., Field J., Cummins A.G. (2011). Capsule endoscopy: a valuable tool in the follow-up of people with celiac disease on a gluten-free diet.. Clin. Transl. Gastroenterol..

[5] Murray J.A., Rubio-tapia A., Van Dyke C.T. (2008). Mucosal atrophy in celiac disease: extent of involvement, correlation with clinical presentation, and response to treatment.. Clin. Gastroenterol. Hepatol..

[6] Mearns E.S., Taylor A., Boulanger T. (2019). Systematic literature review of the economic burden of celiac disease.. PharmacoEconomics.

[7] Ludvigsson J.F., Bai J.C., Biagi F. (2014). Diagnosis and management of adult coeliac disease: Guidelines from the British Society of Gastroenterology.. Gut.

[8] Silvester J.A., Kurada S., Szwajcer A., Kelly C.P., Leffler D.A., Duerksen D.R. (2017). Tests for serum transglutaminase and endomysial antibodies do not detect most patients with celiac disease and persistent villous atrophy on gluten-free diets: A meta-analysis.. Gastroenterology.

[9] Chetcuti Zammit S., Sanders D.S., Sidhu R. (2020). Bone mineral density in patients with celiac disease: A further association with extent of disease on capsule endoscopy.. J. Clin. Gastroenterol..

[10] Chetcuti Zammit S., Sanders D.S., Sidhu R. (2018). A comprehensive review on the utility of capsule endoscopy in coeliac disease: From computational analysis to the bedside.. Comput. Biol. Med..

[11] Al-Toma A., Volta U., Auricchio R. (2019). European Society for the Study of Coeliac Disease (ESsCD) guideline for coeliac disease and other gluten‐related disorders.. United European Gastroenterol. J..

[12] Molder A., Balaban D.V., Jinga M., Molder C.C. (2020). Current evidence on computer-aided diagnosis of celiac disease: Systematic review.. Front. Pharmacol..

[13] Leighton J.A., Triester S.L., Sharma V.K. (2006). Capsule endoscopy: a meta-analysis for use with obscure gastrointestinal bleeding and Crohn’s disease.. Gastrointest. Endosc. Clin. N. Am..

[14] Enns R.A., Hookey L., Armstrong D. (2017). Clinical practice guidelines for the use of video capsule endoscopy.. Gastroenterology.

[15] Delvaux M., Gay G. (2008). Capsule endoscopy: Technique and indications.. Best Pract. Res. Clin. Gastroenterol..

[16] Petroniene R., Dubcenco E., Baker J.P. (2005). Given capsule endoscopy in celiac disease: Evaluation of diagnostic accuracy and interobserver agreement.. Am. J. Gastroenterol..

[17] Biagi F., Rondonotti E., Campanella J. (2006). Video capsule endoscopy and histology for small-bowel mucosa evaluation: A comparison performed by blinded observers.. Clin. Gastroenterol. Hepatol..

[18] Rondonotti E., Spada C., Cave D. (2007). Video capsule enteroscopy in the diagnosis of celiac disease: A multicenter study.. Am. J. Gastroenterol..

[19] Rey J-F., Gay G., Kruse A., Lambert R. (2004). European Society of Gastrointestinal Endoscopy guideline for video capsule endoscopy.. Endoscopy.

[20] Panescu D. (2005). An imaging pill for gastrointestinal endoscopy.. IEEE Eng. Med. Biol. Mag..

[21] Leonard M.M., Silvester J.A., Leffler D. (2021). Evaluating responses to gluten challenge: A randomized, double-blind, 2-dose gluten challenge trial.. Gastroenterology.

[22] Leffler D., Kupfer S.S., Lebwohl B. (2016). Development of celiac disease therapeutics: report of the third gastroenterology regulatory endpoints and advancement of therapeutics workshop.. Gastroenterology.

[23] Ding Z., Shi H., Zhang H. (2019). Gastroenterologist-level identification of small-bowel diseases and normal variants by capsule endoscopy using a deep-learning model.. Gastroenterology.

[24] Byrne M.F., Chapados N., Soudan F. (2019). Real-time differentiation of adenomatous and hyperplastic diminutive colorectal polyps during analysis of unaltered videos of standard colonoscopy using a deep learning model.. Gut.

[25] Kudo S., Misawa M., Mori Y. (2020). Artificial intelligence-assisted system improves endoscopic identification of colorectal neoplasms.. Clin. Gastroenterol. Hepatol..

[26] Kominami Y., Yoshida S., Tanaka S. (2016). Computer-aided diagnosis of colorectal polyp histology by using a real-time image recognition system and narrow-band imaging magnifying colonoscopy.. Gastrointest. Endosc..

[27] Zhou T., Han G., Li B.N. (2017). Quantitative analysis of patients with celiac disease by video capsule endoscopy: A deep learning method.. Comput. Biol. Med..

[28] Siegelman J, Greenblatt E, Galinsky K (2020). A novel ordinal severity scale allows for reproducible assessment of severity of celiac enteropathy by video capsule endoscopy. Presented at Digestive Disease Week 2020 (virtual congress).

[29] Siegelman J., Lewis S., Feuerstein J., Greenblatt E., Bangia U., Leffler D., Mishkin D., Murray J., Yardibi O., Sidhu R. (2019). Assessment of celiac disease severity by video capsule endoscopy with the celiac enteropathy-villous atrophy scale (CE-VASt). Presented at the. International Celiac Disease Symposium.

[30] Tomar S. (2006). Converting video formats with FFmpeg.. Linux J..

[31] He K., Zhang X., Ren S., Sun J. (2016). In Deep residual learning for image recognition, Proceedings of the IEEE conference on computer vision and pattern recognition,.

[32] Cullen N.C., Avants B.B. (2018). Convolutional neural networks for rapid and simultaneous brain extraction and tissue segmentation. Brain Morphometry..

[33] (2021). Image Scoring Using Error Prediction. PCT/JP2021/026607,.

[34] Krippendorff K. (1992). Recent developments in reliability analysis..

[35] Landis J.R., Koch G.G. (1977). The measurement of observer agreement for categorical data.. Biometrics.

[36] Krippendorff K. (2018). Content analysis: An introduction to its methodology; SAGE Publications: NY, USA.

[37] Koh J.E.W., Hagiwara Y., Oh S.L. (2019). Automated diagnosis of celiac disease using DWT and nonlinear features with video capsule endoscopy images.. Future Gener. Comput. Syst..

[38] Hamza R., Muhammad K., Lv Z., Titouna F. (2017). Secure video summarization framework for personalized wireless capsule endoscopy.. Pervasive Mobile Comput..

[39] Maiden L., Elliott T., McLaughlin S.D., Ciclitira P. (2009). A blinded pilot comparison of capsule endoscopy and small bowel histology in unresponsive celiac disease.. Dig. Dis. Sci..

[40] Spada C., Riccioni M.E., Urgesi R., Costamagna G. (2008). Capsule endoscopy in celiac disease.. World J. Gastroenterol..

