# Supplementary material for: Quantification of Celiac Disease Severity Using Video Capsule Endoscopy: A Comparison of Human Experts and Machine Learning Algorithms
Source: Curr Med Imaging. 2023 Jun 2;19(12):1455–62. doi: 10.2174/1573405619666230123110957 (PMC10364343; doi:10.2174/1573405619666230123110957)
Supplement: Supplementary file 1 [file CMIM-19-1455_SD1.pdf]

Supplementary Material

Quantification of Celiac Disease Severity Using Video Capsule Endoscopy: A Comparison of Human Experts and Machine Learning Algorithms

Stefania Chetcuti Zammit[1,*], Mark E. McAlindon[1], Elliot Greenblatt[2], Michael Maker[2], Jenifer Siegelman[3], Daniel A. Leffler[3], Ozlem Yardibi[3], David Raunig[3], Terry Brown[3] and Reena Sidhu[1,4]

[1]Academic Unit of Gastroenterology and Hepatology, Sheffield Teaching Hospitals NHS Hospital Trust, Sheffield, UK; [2]Invicro, a Konica Minolta Company, Boston, MA, USA; [3]Takeda Pharmaceuticals Inc. Co., Cambridge, MA, USA; [4]Department of Infection, Immunity and Cardiovascular Disease, University of Sheffield, Sheffield, UK

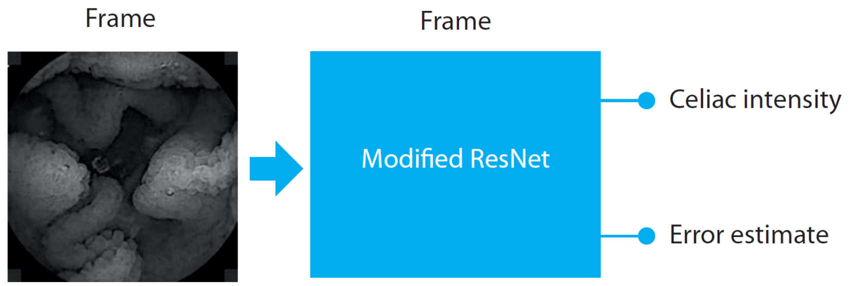

Supplementary Figure 1. A visual illustration of the machine learning algorithm.

The machine learning algorithm (MLA) consisted of a modified residual network (ResNet) convolutional neural network architecture that was fed grayscale versions of the frames with the position of the frame along the small intestine [1]. The MLA was trained using an independent training data set of 334 080 frames (35 videos) from patients with biopsy-proven celiac disease (CeD), who underwent video capsule endoscopy (VCE) as part of their clinical care. Additionally, 110 579 frames (13 videos) from patients without CeD who underwent VCE for unrelated symptoms were also included in the training data. The MLA was further modified to predict both disease severity and an error estimate of the frame prediction. Each frame was read by the MLA in eight different orientations, and the predictions were averaged using the inverse of the error estimates squared to weight each orientation. The architecture was implemented in Python v3.6 (Python Software Foundation, Wilmington, DE, USA) using ANTsPyNet and TensorFlow as the backend [2]. Videos had a mean number of 8300 frames and were machine analyzed in 15 minutes using a single NVIDIA® V100 graphics processing unit (NVIDIA® Corporation, Santa Clara, CA, USA), with 32 gigabytes of dedicated random-access memory on a NVIDIA® DGX workstation.

(a)

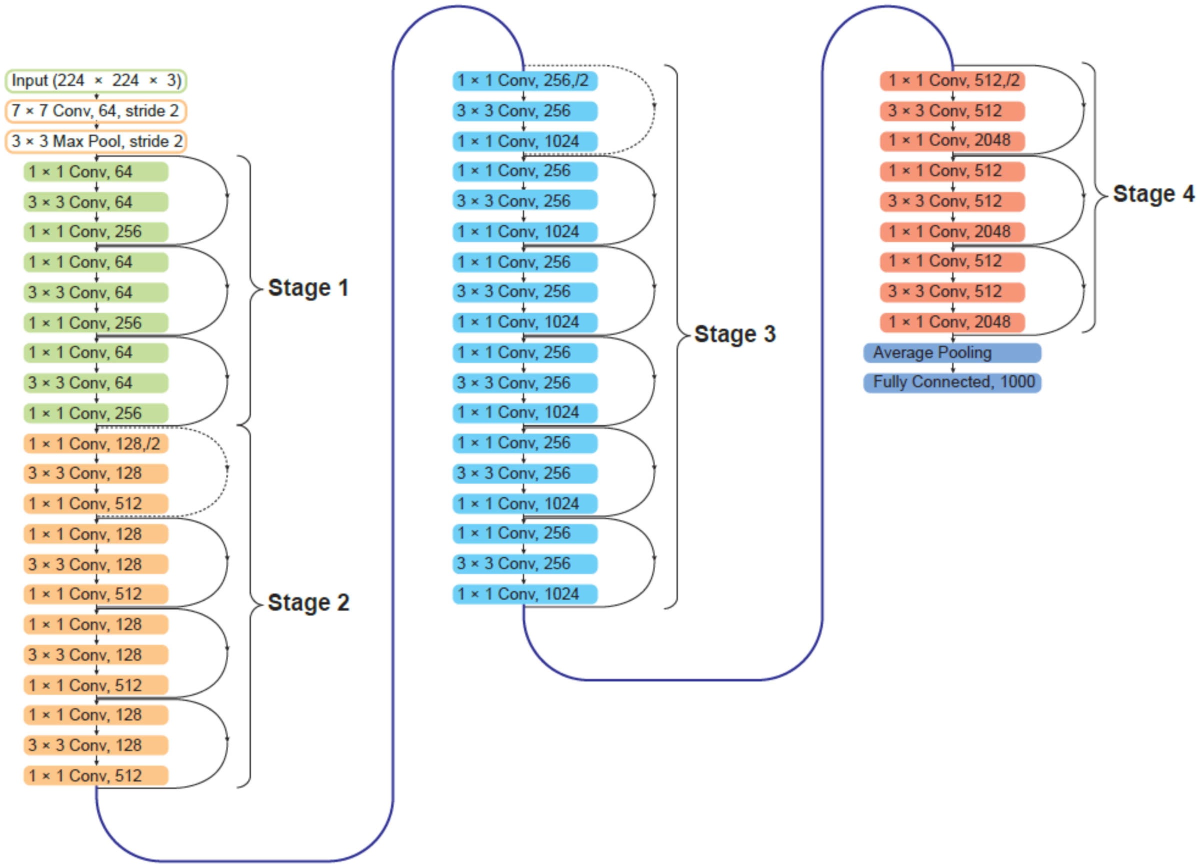

(b)

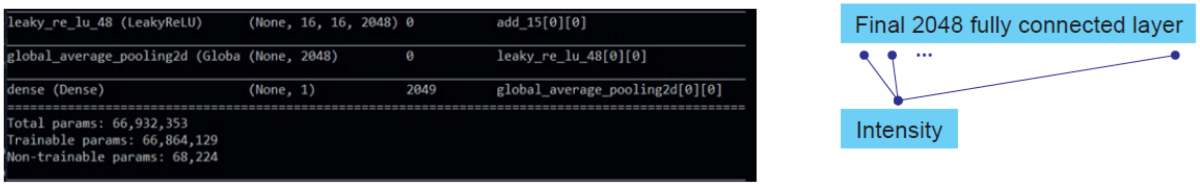

(c)

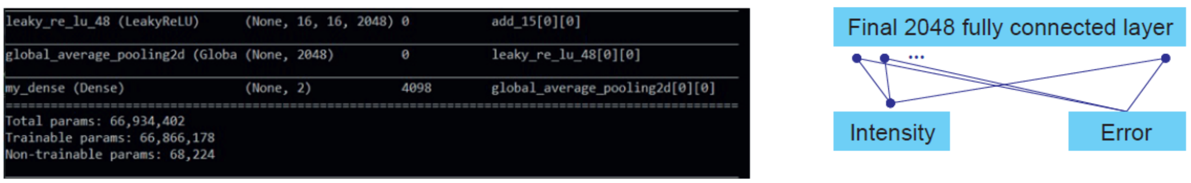

Supplementary Figure 2. Modification of the ResNet network during algorithm development.

(a) Traditional ResNet50 network structure: We modified the network to accept 512 × 512 input, use 64 3 × 3 base filters, and use a 2048 final fully connected layer. We also used sigmoid activation for the final fully connected layer.

(b) Final layers of the ResNet50 network, adapted to 512 × 512 input and using 64 3 × 3 base filters, with a 2048 fully connected final layer.

(c) Final layers of the ResNet50 network after adding a second output node connected to the same 2048 fully connected layer. The addition of the error output creates a ResNet network identical to one trained to regress two values rather than one only. This was achieved by adding a second output node connected to the final 2048 layer, which adds 2048 weight and a bias. The network has 67 million trainable parameters. ResNet, residual network.

Supplementary Text 1. Methodology for the assessment of association between video capsule endoscopy findings and clinical parameters.

Clinical data were extracted by one gastroenterologist (S.C.Z.) from digital clinical letters, endoscopy, and blood reporting databases within the Sheffield Teaching Hospitals NHS Foundation Trust, UK within 4 months of video capsule endoscopy (VCE) being performed. Data collected included patient serology (tissue transglutaminase [tTG], albumin, hemoglobin, vitamin B12, folate, vitamin D, and calcium), biopsy data from esophago-gastro-duodenoscopy, patient human leukocyte antigen phenotype, and refractory celiac disease (CeD) status. Serological and histological outcomes (based on Marsh scores) were also gathered as part of physician global assessments (PGAs). PGA parameters evaluated included symptoms at VCE, malabsorption (assessed using patient hemoglobin, albumin, vitamin B12, and folate levels), levels of anti-tTG and intraepithelial lymphocytes, and the extent of villous atrophy (Supplementary Table **1**). Celiac time (the amount of time during which signs of CeD in the small intestine were observed using VCE) was also collected. Celiac time fraction was then calculated (celiac time/total small intestine transit time). Data regarding the presence of endomysial antibodies (EMAs), and genetic and refractory status were also collected.

Expert reader average VCE parameters were compared with available clinical measures using Pearson's Rho (continuous clinical data; e.g. serology), Spearman's Rho (ordinal data; e.g. PGA scores), and one-way ANOVA *P* values (categorical data; e.g. refractory status). Comparison of VCE parameters between patient groups (based on the presence of EMAs and genetic and refractory status) was conducted using one-way ANOVA *P* values.

Supplementary Text 2. Results of assessment of association between video capsule endoscopy assessment results and clinical parameters.

Patient serology

Overall, a weak positive correlation was observed between patient serologic data and expert reader VCE scores (Rho < 0.44; Supplementary Table **3**). Weakly negative and mixed correlations were observed for hemoglobin and folate with VCE parameters, respectively, and a weakly negative correlation was present for vitamin B12. All measures of VCE data demonstrated a negative correlation with patient calcium levels (−0.22 < Rho < −0.44), albumin (−0.07 < Rho < −0.28), and vitamin D (−0.09 < Rho < −0.28), and anti-tTG showed a positive correlation with first tertile measures (0.22 < Rho < 0.30).

Physician global assessment and histologic findings

Mixed correlations were observed between PGA scoring, histology, and expert reader VCE scores (Supplementary Table **4**). The average of the scored frames in the first 5% of VCE videos showed the strongest positive correlations with PGA and histologic outcomes, although negatively correlated with patient symptoms and the presence of malabsorption. Marsh scores for the duodenal bulb (D1) and the second part of the duodenum (D2) correlated positively with VCE measures (0.47 < Rho < 0.65 and 0.31 < Rho < 0.51, respectively). Histology (as measured by PGA) showed a weaker correlation with VCE measures (0.24 < Rho < 0.43) than Marsh scores. Overall, results from PGAs showed some correlation with the findings from VCE (0.19 < Rho < 0.31). Malabsorption showed no correlation with VCE findings, and symptoms demonstrated a weak negative correlation with these data.

Endomysial antibodies, genetic, and refractory status

Examining patients when grouped according to EMA levels or human leukocyte antigen DQ2 alleles did not result in substantial VCE differences between patient groups (Supplementary Table **5**). In total, nine patients had a refractory status of 1 and three patients had a refractory status of 2. Patients with refractory disease had significant differences ($P < 0.05$) in all measures except the first 5% mean ($P = 0.0579$) compared with the other patient groups evaluated, with differences most apparent for the mean for the entire small intestine and celiac time.

Supplementary Table 1. Physician global assessment for celiac disease.

Characteristic	Severity		
	0	1	2
Symptoms at capsule endoscopy*	None/mild/atypical	Moderate classic	Severe classic
Malabsorption[†]	None/mild	Moderate	Severe
Serology (anti-tTG U/mL 0–7)	< 2 × ULN	> 2 × ULN	NA
Histology	Normal/increased IELs/crypt hyperplasia (Marsh score 0–2)	Patchy VA (Marsh score 3a/3b)	Severe VA (Marsh score 3c)/evidence of RCD (monoclonality, ulcerative jejunitis etc.)

Overall scoring: remission: 0–1; mild: 2–4; moderate/severe: 5–6.
*Symptoms were scored according to the number experienced: mild, 0–2; moderate, 3–4; and severe, > 5.
[†]Malabsorption was defined as the presence of anemia, low albumin with/without vitamin B12/folate deficiency. If albumin and hemoglobin were in different categories, it was advised to consider the most severe category. Clinical markers considered as part of malabsorption assessment were: hemoglobin (mild, 110–119 g/L; moderate, 80–109 g/L; severe, < 80 g/L), albumin (normal, 35–45 g/dL; moderate, 25–35 g/dL; severe, < 25 g/dL), vitamin B12 (deficient, < 197 ng/L), and folate (deficient, < 3.9 µg/L).
IEL, intraepithelial lymphocyte; NA, not applicable; RCD, refractory celiac disease; tTG, tissue transglutaminase; ULN, upper limit of normal; VA, villous atrophy.

Supplementary Table 2. Patient serology and Marsh scores at video capsule endoscopy.

Serologic Parameter	Value for Included Patients at VCE (N = 63)
**Albumin (g/dL)**	
Mean (SD)	46.2 (3.7)
Median	47
**Hemoglobin (g/L)**	
Mean (SD)	138.0 (16.6)
Median	137.0
**CRP (mg/L)**	
Mean (SD)	3.2 (9.5)
Median	0.7
**Vitamin B12 (ng/L)**	
Mean (SD)	476.0 (340.0)
Median	384.0
**Folate (µg/L)**	
Mean (SD)	9.1 (6.3)
Median	6.4
**Vitamin D (nmol/L)**	
Mean (SD)	68.0 (35.2)
Median	63.1
**Calcium (mmol/L)**	
Mean (SD)	2.3 (0.1)
Median	2.3
**Anti-tTG (U/mL)**	
Mean (SD)	49.1 (48.8)
Median	30.0
**EMAs***	45
Patients with positive result, n	
**Marsh score D1**	
Mean (SD)	3.4 (1.5)
Median	4.0
**Marsh score D2**	
Mean (SD)	4.0 (1.4)
Median	4.0

*Patients were evaluated for the presence of EMA antibodies.
CRP, C-reactive protein; D1, duodenal bulb; D2, second part of the duodenum; EMA, endomysial antibody; SD, standard deviation; tTG, tissue transglutaminase; VCE, video capsule endoscopy.

Supplementary Table 3. Correlation between expert reader video capsule endoscopy scores and patient serologic data as shown by Pearson's Rho values.

Parameter	Anti-tTG (n = 56)	Albumin (n = 62)	Hb (n = 60)	Vitamin B12 (n = 60)	Folate (n = 60)	Vitamin D (n = 57)	Calcium (n = 60)
First tertile mean	0.23	−0.18	−0.11	−0.11	0.07	−0.14	−0.41
First tertile maximum	0.29	−0.27	−0.09	−0.10	0.00	−0.11	−0.43
Mean value over the entire small intestine	0.06	−0.17	−0.13	−0.11	0.17	−0.12	−0.32
Mean score from the first 5% of VCE video	0.17	−0.19	−0.07	−0.11	−0.03	−0.14	−0.33
Celiac minutes	−0.03	0.00	−0.11	−0.09	0.18	−0.18	−0.23
Celiac time fraction	0.00	−0.08	−0.18	−0.08	0.17	−0.10	−0.23

'n' represents the number of patients with clinical data available. Celiac minutes was defined as the time the VCE capsule witnessed evidence of celiac disease. Celiac time fraction was calculated by dividing celiac minutes by the total small intestinal transit time.
Hb, hemoglobin; tTG, tissue transglutaminase; VCE, video capsule endoscopy.

Supplementary Table 4. Correlation among results of the expert reader evaluation of video capsule endoscopy data, physician global assessment, and histologic data.

Parameter	Pearson's Rho Value							
	Symptoms at VCE (n = 63)	Malabsorption (n = 61)	Serology (n = 62)	Histology (n = 55)	Overall PGA Score (n = 55)	Marsh Score D1 (n = 52)	Marsh Score D2 (n = 55)	Marsh Score Maximum (n = 57)
First tertile mean	−0.14	−0.12	0.21	0.27	0.21	0.50	0.35	0.27
First tertile maximum	−0.17	−0.08	0.22	0.25	0.20	0.48	0.35	0.24
Mean value over the entire small intestine	−0.15	−0.11	0.22	0.27	0.21	0.50	0.36	0.29
Mean score from the first 5% of VCE video	−0.24	−0.04	0.21	0.33	0.27	0.64	0.50	0.49
Celiac time	−0.08	−0.14	0.17	0.42	0.29	0.52	0.32	0.38
Celiac time fraction	−0.11	−0.09	0.18	0.40	0.30	0.52	0.33	0.38

'n' represents the number of patients with clinical data available.
D1, duodenal bulb; D2, second part of the duodenum; PGA, physician global assessment; VCE, video capsule endoscopy.

Supplementary Table 5. Correlation among results of the expert reader evaluation of video capsule endoscopy data and findings on endomysial antibodies and genetic and refractory status.

Parameter	*P* value		
	EMAs Present (n = 59)	DQ2 Homozygous (n = 41)	Refractory Disease (n = 63)
First tertile mean	0.66	0.17	0.007
First tertile maximum	0.85	0.38	0.022
Mean value over the entire small intestine	0.06	0.19	< 0.001
Mean score from the first 5% of VCE video	0.94	0.43	0.058
Celiac minutes	0.07	0.16	< 0.001
Celiac time fraction	0.06	0.11	< 0.001

'n' represents the number of patients with clinical data available.
EMA, endomysial antibody; VCE, video capsule endoscopy.

REFERENCES

[1] Xie, S.; Girshick, R.; Dollár, P.; Tu, Z.; He, K. Aggregated residual transformations for deep neural networks, Proceedings of the IEEE Conference on Computer Vision and Pattern Recognition, 2017; pp 1492–1500.

[2] Cullen, N. C.; Avants, B. B., Convolutional neural networks for rapid and simultaneous brain extraction and tissue segmentation. In *Brain Morphometry*, Springer: 2018; pp 13–34.
